# A Culturally Sensitive Social Support Intervention for Chinese American Breast Cancer Survivors (Joy Luck Academy): Protocol for a Randomized Controlled Trial

**DOI:** 10.2196/30950

**Published:** 2021-09-22

**Authors:** Qian Lu, Krystal Warmoth, Lingjun Chen, Christine S Wu, Qiao Chu, Yisheng Li, Matthew W Gallagher, Annette L Stanton, Marjorie Kagawa Singer, Lucy Young, Alice Loh

**Affiliations:** 1 Department of Health Disparities Research The University of Texas MD Anderson Cancer Center Houston, TX United States; 2 Department of Psychology University of Houston Houston, TX United States; 3 Department of Biostatistics The University of Texas MD Anderson Cancer Center Houston, TX United States; 4 Department of Psychology University of California Los Angeles Los Angeles, CA United States; 5 Department of Psychiatry and Biobehavioral Sciences University of California Los Angeles Los Angeles, CA United States; 6 Department of Community Health Sciences University of California Los Angeles Los Angeles, CA United States; 7 Herald Cancer Association San Gabriel, CA United States

**Keywords:** social support, peer mentor support, randomized controlled trial, Chinese cancer survivors, breast cancer

## Abstract

**Background:**

Breast cancer is the most prevalent type of cancer among Asian American women. Chinese American immigrant breast cancer survivors face unique challenges because of cultural and socioecological factors. They report emotional distress and the need for social, emotional, and spiritual support. However, culturally and linguistically appropriate information for managing survivorship health care is often unavailable.

**Objective:**

To improve the health outcomes for this underserved and understudied population, we developed, designed, and launched a randomized controlled trial to test the health benefits of a culturally sensitive social support intervention (Joy Luck Academy). In this paper, we describe the research protocol.

**Methods:**

This randomized controlled trial will enroll Chinese-speaking, stage 0 to 3 breast cancer survivors who have completed treatment within the previous 36 months using a community-based participatory research approach. We will randomly assign 168 participants to the intervention or control group. The intervention arm will attend 7 weekly 3.5-hour peer mentor and educational sessions. The control group will receive the educational information. We will assess health outcomes at baseline, immediately after the Joy Luck Academy, and at 1- and 4-month follow-ups. The primary outcome is quality of life, as measured by the Functional Assessment of Cancer Therapy scale. Secondary outcomes include depressive symptoms, positive affect, fatigue, and perceived stress. We will also explore how the intervention influences cortisol levels. To identify how and to whom the program is effective, we will measure social and personal resources and theorized mechanisms and perform qualitative interviews with a subsample of participants to enhance the interpretation of quantitative data.

**Results:**

Recruitment began in February 2015, and data collection was completed in February 2019. We expect to complete data management by August 2021 and publish results in 2022.

**Conclusions:**

If the Joy Luck Academy is demonstrated to be effective, it may be easily disseminated as an intervention for other groups of Asian American immigrant breast cancer survivors. Furthermore, similar programs could be integrated into other diverse communities.

**Trial Registration:**

ClinicalTrials.gov NCT02946697; http://clinicaltrials.gov/ct2/show/NCT02946697.

**International Registered Report Identifier (IRRID):**

DERR1-10.2196/30950

## Introduction

### Background

Breast cancer is the most prevalent type of cancer among Asian American women, and its incidence is increasing [1,2]. Higher survival rates have led to a greater focus on improving cancer survivors’ quality of life [3,4]. Asian American immigrant breast cancer survivors continue to face unique challenges because of cultural and socioecological factors [5,6]. Although they commonly report emotional distress and express the need for social, emotional, and spiritual support [7], they are less likely to seek support from family, friends, or mental health professionals than White Americans [8]. Asian American breast cancer survivors report cultural stigma toward cancer, gender role socialization as *caregivers*, and fear of burdening family as barriers to seeking support [6-9]. In addition, they lack culturally and linguistically appropriate information to manage their survivorship health care [7,10]. Limited English proficiency also limits survivors’ ability to communicate with health care providers, understand health-related information, and make decisions about survivorship care [7]. Thus, Asian American breast cancer survivors have various informational and psychosocial needs that are not addressed by existing evidence-based interventions.

Psychosocial interventions delivered after the completion of primary oncologic treatment have improved the quality of life of breast cancer survivors [11,12]. A systematic review and meta-analysis of different psychosocial interventions showed improvement in emotional distress, anxiety, depression, and quality of life after treatment [12]. For example, women undergoing educational and nutritional interventions showed fewer depressive symptoms and improved physical functioning at follow-up [13]. Psychosocial interventions (supportive, expressive discussion groups) reduced loneliness, promoted hope, and enhanced the quality of life in women with breast cancer [14]. In addition, women who lacked personal resources (eg, self-esteem) or social support were more likely than those with these resources to show physical health improvements after participating in a psychosocial intervention [15].

Most previous interventions on breast cancer survivorship have been conducted among English-speaking, highly educated White women. Sociocultural differences between White and Asian American immigrant women may limit the applicability of existing interventions to Asian American breast cancer survivors. For example, 50% of Asian Americans do not speak fluent English [16]; however, existing interventions do not address language barriers in communication between patients and health care providers. Moreover, existing interventions do not target cultural factors that affect the quality of life, service use, or support-seeking attitudes among Asian American cancer survivors. Hence, although psychosocial interventions confer significant benefits to health and well-being, existing interventions may not address the specific needs of Asian American breast cancer survivors.

The Joy Luck Academy program was developed for Chinese-speaking breast cancer survivors [17], using a community-based participatory research (CBPR) approach (ClinicalTrials.gov NCT02946697) [18-21]. The Joy Luck Academy includes two components: education and peer mentor support. Each component was chosen based on the needs of this group, including lack of knowledge about breast cancer and survivorship management, feelings of loneliness, lack of emotional support, communication difficulties, and body image concern [22].

The results of a pilot study suggested that Joy Luck Academy has the potential to improve well-being among Chinese American breast cancer survivors [17,23]. Furthermore, qualitative data revealed that Joy Luck Academy reduced perceived stigma and loneliness and increased a sense of belonging by providing a forum for participants to share their experiences with women of similar cultural backgrounds [17]. The pilot study results confirmed that the intervention was feasible and valued by Chinese American breast cancer populations and that the CBPR approach improved the cultural sensitivity of the intervention.

### Objectives

We planned a randomized controlled trial (RCT) to test the health benefits of Joy Luck Academy and identify how and to whom the Joy Luck Academy is effective using a CBPR approach and mixed methods. This study is the first RCT to test a culturally and linguistically sensitive intervention designed to address the informational and emotional needs of Chinese American breast cancer survivors.

The primary aim of this study is to test the health benefits of a culturally sensitive social support program for Chinese American breast cancer survivors (aim 1). We hypothesize that the program will confer health benefits, as indicated by improvements in outcomes. The primary outcome is quality of life; the secondary outcomes are depressive symptoms, fatigue, positive affect, and perceived stress. We also expect that the intervention will normalize diurnal cortisol levels, an exploratory outcome. Secondary objectives include identifying the characteristics of individuals who benefit from the Joy Luck Academy (aim 2) and understanding the underlying mechanisms (aim 3). On the basis of the literature and our pilot study [15,17], we hypothesize that Joy Luck Academy will be more effective for women who lack psychosocial resources, specifically, those with low levels of social support, optimism, and perceived control over illness. We hypothesize that the Joy Luck Academy will increase relatedness need satisfaction and coping self-efficacy and decrease cancer-related perceived stigma, leading to health benefits (ie, mediators of the intervention effect).

## Methods

### Overview

We will conduct an RCT in Chinese American breast cancer survivors who have completed primary treatment. Participants will be randomly assigned to the Joy Luck Academy intervention or control group. Health outcomes will be assessed at baseline and immediately after the intervention (7 weeks after baseline) and at 1 and 4 months after completing the Joy Luck Academy. All study materials will be in Chinese.

### Participants

We will recruit 168 Chinese American breast cancer survivors, as determined by a sample size calculation (described below in *Power Analysis* section). Experienced researchers and community staff who speak fluent Chinese (Cantonese or Mandarin) will introduce potential participants to the study, screen them for eligibility, and invite them to participate; if they decline to participate, their reasons for declining will be documented. Upon completion of the study, each participant will be given prorated US $180 in gift cards as compensation.

### Eligibility Criteria

The inclusion criteria were (1) self-identifying as being comfortable speaking Mandarin or Cantonese, (2) women having stage 0 to 3 breast cancer, and (3) having completed primary treatment (surgery, chemotherapy, or radiotherapy) within the previous 36 months.

### Recruitment

Participants will be recruited from the Greater Los Angeles area in Southern California. We will identify and recruit potential participants via a community organization (Herald Cancer Association [HCA]) and local advertising. The HCA provides many community services, including providing Chinese-language cancer information brochures. The HCA maintains a large database of primarily Chinese immigrant breast cancer survivors, who registered with the HCA to obtain educational and informational resources. The HCA will contact individuals on their client list, advertise the study in their monthly newsletters, ask for referrals from participants, and promote the study at cancer survivor events. Targeted announcements will be used in the Chinese American community to reach women who are not in the HCA database. The San Gabriel Valley region of Los Angeles County has the largest concentration of Chinese American communities in the United States [24]. Announcements will be distributed to local clinics, doctors’ offices, and other patient services.

### Consent

We will obtain approval from the human subjects protection committees of the University of Houston (Houston, Texas), the University of Texas MD Anderson Cancer Center (Houston, Texas), and the California Cancer Registry (Sacramento, California) to conduct this RCT. Written informed consent will be required before participation from each participant and will be obtained by the HCA.

### Power Analysis

Power analysis was based on the primary aim of this trial. Specifically, we calculated the sample size required to detect the effect of Joy Luck Academy on the primary outcome (quality of life at the 4-month follow-up assessment). In our primary analyses, we will use maximum likelihood-type (eg, restricted maximum likelihood) estimation procedures based on all observed data, assuming a missing-at-random mechanism [25]. The pilot study revealed that the Joy Luck Academy had an intermediate or higher effect on improving participants’ quality of life (Cohen *d*=0.47), depressive symptoms (Cohen *d*=0.55), and positive affect (Cohen *d*=0.62). On the basis of a targeted medium effect size of Cohen *d*=0.47, we estimated that a total sample size of 146 women at the 4-month follow-up would yield 80% power to detect this treatment effect at α=.05 (nQuery Advisor Version 7.0, Statistical Solutions Ltd). Assuming an attrition rate of 10%-20%, the final recruitment goal will range between 162 and 183 at the baseline.

### Randomization

Participants who consent to participate will be randomly assigned to the Joy Luck Academy or control group at an approximate 1:1 ratio. Using minimization—a covariate adaptive randomization approach [26]—we will assign a participant to the intervention or control group by applying a randomization algorithm that will take into account participants’ age, stage of cancer, and time since completing treatment. This covariate information will be obtained by the HCA staff during the participant screening process. Randomization will start 4 weeks before the orientation scheduled start date and continue until the recruitment goal is reached or until 1 week before the start of each cohort study. Only the researcher conducting random assignments and the person who informs participants of their group allocation will be aware of the condition assignment.

### Intervention Group

Each Joy Luck Academy intervention program will enroll a maximum of 24 participants. We plan to deliver seven Joy Luck Academy programs during the project period. The Joy Luck Academy programs will have the same instructors, lectures, and support materials to ensure the fidelity of programs delivered at different times.

Joy Luck Academy participants will meet for 3.5 hours once a week for 7 consecutive weeks. Each session will begin with a 30-minute breakfast to allow for informal conversations among participants (mentees) and mentors. The meal is followed by a lecture given by the instructors on cancer-related topics and a question-answer session. After the lecture, 15 minutes of physical exercise will be engaged, followed by a 15-minute break. After the break, participants will join class activities and group sharing in the format of a large group sharing led by a Joy Luck Academy program facilitator, a small group sharing led by peer mentors, or a combination of both. The lecture, question-answer, class activities, and group sharing take about 2.5 hours in total.

Joy Luck Academy lecture topics include recognizing the side effects of treatment and differentiating them from the symptoms of cancer recurrence, physical therapy and complementary treatments, stress management, recognizing depression and managing emotional problems, communication with family members, and body image. The Joy Luck Academy instructors are professionals who are experienced in breast cancer treatment and support, including a breast surgeon, a clinical psychologist, a physical therapist, a dietitian, and a beautician. They will give lectures in their areas of expertise and answer the Joy Luck Academy participants’ questions. Some sessions will be audio recorded to determine fidelity to the protocol, lecture plan, and activity objectives.

Each peer mentor will work with 3 to 5 mentees. Peer mentors will lead small group discussions on that week’s topic and share their own experiences with mentees. This setup will encourage and allow mentees to share personal feelings in a relaxed and comfortable setting and receive support and advice. Mentors will also contact mentees once a week during the 7-week program to provide guidance and address remaining concerns. Each participant will be assigned to a volunteer peer mentor who is a breast cancer survivor and speaks a similar Chinese dialect. Mentors will be recruited from graduates of Joy Luck Academy pilot programs. Each potential mentor will be evaluated by the program facilitator based on the potential mentor’s engagement in the program and support of other participants during the program. Mentors will complete a 3-hour training program to gain mentoring skills. The training will be conducted by HCA staff using a standardized manual that was jointly developed by the academic and community teams. The training program focuses on skills in establishing rapport, demonstrating understanding, listening empathically, discussing participants’ concerns, and promoting the sharing of information and feelings. The training also involves lectures, case study presentations, discussions, and role-play activities.

We will use a procedure to maximize attendance and retention recommended by a previous study [27]. Participants will be asked to notify the program facilitator or their assigned mentor in advance if they will be absent. They will be told that, with their permission, the group members will be informed of the reason for their absence to reduce worries and concerns. The mentor will keep copies of the handouts for missing participants and call them within 5 days to briefly discuss the week’s topic and tell them that they can pick up the handouts when they return to class. This should make participants feel that they are cared for by others and that they can catch up and will make them more likely to continue the program. Participants will also be informed that the lectures, recorded in DVD format, will be available for them to view at home so that they will not miss information in the lecture.

### Control Group

Control group participants will undergo usual care and receive information booklets in Chinese that were developed by the American Cancer Society [28]. The booklets cover common issues related to breast cancer, various treatments, and life after treatment, which are similar to the topics covered in the Joy Luck Academy. Control group participants will be informed that they are in the self-study group to read the information booklets on their own to reduce potential anticipation bias and they will have the opportunity to join an in-person study group (Joy Luck Academy) after the assessment at the 4-month follow-up.

### Data Management and Confidentiality

All participants’ identification records will be kept confidential and stored in a secure file with password protection. Each participant will be assigned an identification number that will be used on all documents and data files. All protected health information will be removed from the data when it is exported for analyses.

### Data Collection

#### Overview

After consenting, participants will complete the baseline questionnaires during an orientation session. These measures will be used to assess participants’ demographic and treatment information and psychosocial variables of interest. Participants’ demographic information (age, income, education level, and length of time living in the United States) and acculturation level [29] will be self-reported. Cancer and treatment information (cancer stage, treatments, and time since diagnosis) will be self-reported and confirmed through medical records, with participants’ consent.

Self-reported health outcomes and mediators will be measured via questionnaires at baseline, immediately after Joy Luck Academy completion and 1 month and 4 months after the intervention. The hypothesized moderating variables will only be measured at the baseline. The questionnaires, along with preaddressed, postage-paid envelopes, will be mailed to all participants. Participants will be asked to complete the questionnaires at home within 7 days and return them by mail. They will be reminded by phone to complete the questionnaires.

#### Primary Outcome

Quality of life during the previous week will be measured using the Chinese version [30] of the Functional Assessment of Cancer Therapy-Breast (FACT-B) [31]. FACT-B comprises four Functional Assessment of Cancer Therapy-General (FACT-G) subscales (physical, social, emotional, and functional well-being) and an additional subscale pertinent to breast cancer. Each item is rated on a 5-point scale (0=not at all, 1=a little bit, 2=somewhat, 3=quite a bit, and 4=very much). To improve the comparison between this and other trials, the FACT-G total score (the sum of all the items in the first four subscales) will be used as the primary outcome. The breast cancer concern subscale will be used as an exploratory outcome measure. Higher FACT-G scores indicate a better quality of life.

#### Secondary Outcomes

Depressive symptoms will be measured with the Chinese short-form version [32] of the Center for Epidemiologic Studies Depression (CES-D) scale [33]. Two additional items (“I don’t want to have contact with people, socialize, or go out at all” and “I have thought about hurting myself”) from the Chinese American Depression Scale, which was specifically developed for Chinese Americans [34] to capture depressive symptoms that are not assessed in the CES-D, will be used as an exploratory outcome measure. Participants will indicate on a 4-point scale how often they felt or behaved in a given way during the previous week (0=rarely or none of the time [less than a day], 1=some or a little of the time [1-2 days], 2=occasionally or a moderate amount of the time [3-4 days], and 3=most of the time [5-7 days]). Higher total scores indicate more depressive symptoms.

Positive affect will be assessed using the 10 positive affect items (eg, *cheerful*) of the Positive and Negative Affect Scale [35]. Participants will indicate to what extent they have generally felt an emotion (very slightly or not at all, a little, moderately, quite a bit, or extremely) during the previous week on a 5-point scale, which is summed. Higher scores indicate a more positive affect.

Fatigue will be assessed using 6 items of the Functional Assessment of Chronic Illness Therapy-Fatigue scale [36]. The items were selected based on our pilot studies. Participants will indicate how true each statement (eg, “I feel tired”) had been for them during the previous week on a 5-point scale (0=not at all, 1=a little bit, 2=somewhat, 3=quite a bit, and 4=very much). Higher scores on the summed 6 items indicate greater fatigue.

Perceived stress will be measured using a 4-item short version of the Perceived Stress Scale [37]. Participants will be asked how often they experienced particular feelings and thoughts during the previous week (eg, “I was unable to control important things in my life”) on a 5-point scale (0=not at all, 1=a little bit, 2=somewhat, 3=quite a bit, 4=very much). The four items are summed, with higher scores indicating greater perceived stress.

#### Exploratory Outcomes

Salivary cortisol levels will be assessed at baseline and immediately after the intervention. Participants will collect saliva 20 minutes after awakening and at 12 PM, 5 PM and 9 PM hours on 2 consecutive days so that both the mean level and slopes can be calculated [38]. Normal diurnal rhythms are expected to consistently demonstrate peak cortisol concentrations during awakening and decline thereafter [39]. Following established procedures [39,40], participants will be given detailed instructions on collecting saliva at home. Participants will be asked not to eat or drink anything, brush or floss their teeth, use mouthwash or lipstick, or smoke 30 minutes before collection. They will complete questions on comorbid conditions (such as autoimmune disorders) and medications (such as prednisone, dexamethasone, and other steroids), alcohol and caffeine intake, exercise level, and sleep quality, which influence cortisol levels. They will be given salivettes and then asked to chew a small cotton pad and spit it back into the tube of the salivette. Participants will be asked to mail their samples to the HCA office within 3 days after collection (salivary cortisol is stable at ambient temperature for 2-4 weeks) [40]. The samples will be stored in a freezer at −20°C until they are delivered in batches to a well-established independent laboratory for analysis; these procedures are routinely used [40].

#### Mediating Variables

Relatedness need satisfaction will be assessed using the eight-item relatedness subscale of the General Need Satisfaction Scale [41]. Participants will be asked to think about how each item (eg, “People in my life care about me”) relates to their life and indicate how true it has been for them in the previous week on a scale from 1 (not true at all) to 7 (very true). The eight summed items indicate the extent to which relatedness needs are satisfied. Higher scores indicate higher needs satisfaction for relatedness.

Perceived stigma will be measured using the modified nine-item Self-Stigma Scale-Short Form [42]. The scale was originally developed for minorities and was modified for breast cancer in this study. Participants will rate how each item (eg, “I fear that others would know that I am a breast cancer survivor”) is related to their thoughts and feelings about being a breast cancer survivor on a 4-point scale (1=strongly disagree, 2=disagree, 3=agree, and 4=strongly agree). Higher summed scores indicate greater perceived self-stigma.

Coping self-efficacy will be assessed using 19 items selected from the Cancer Behavior Inventory [43], a measure of self-efficacy for coping with cancer or cancer treatment. Participants will rate their confidence in accomplishing a behavior (eg, “sharing feelings of concerns”) on a scale from 1 (not confident at all) to 9 (totally confident). Higher scores on the summed 19-item scale indicate greater self-efficacy.

#### Moderating Variables

Social support will be measured using two subscales (positive interactions and emotional and informational support) of the Medical Outcomes Study Social Support Survey [44], a multidimensional instrument that is valid in Chinese-speaking patients [45]. Each item is rated on a 5-point scale (1=none of the time, 2=a little of the time, 3=some of the time, 4=most of the time, 5=all of the time). The summed subscale scores include eight items for emotional and informational support and four items for positive interactions. The overall scale score is the sum of the two subscales, with higher scores indicating greater social support.

Dispositional optimism will be assessed using the Chinese Revised Life Orientation Test [46], which comprises three positively worded (eg, “I am always optimistic about my future”) and three negatively worded (eg, “I hardly ever expect things to go my way”) phrases. Each item is rated on a 4-point scale (1=disagree, 2=neutral, 3=agree, and 4=strongly agree). Higher summed scores indicate higher optimism.

Perceived control of illness will be measured using three items previously used in a psychosocial intervention with breast cancer survivors [15]. These items assess perceived control of the future course of illness, day-to-day symptoms, and emotions related to illness, with each rated on a 4-point scale (0=none, 1=a little bit, 2=somewhat, and 3=a lot). Higher scores on the summed items indicate greater perceived control.

The FACT-B, CES-D, Self-Stigma Scale, Chinese Revised Life Orientation Test, Perceived Stress Scale, and Medical Outcomes Study Social Support Survey have been validated in Chinese populations, and the other scales have been or will be translated into Chinese and back-translated into English by bilingual researchers through an iterative process to ensure conceptual and linguistic equivalence. Most of the Chinese versions have been used in previous studies [47] and have demonstrated good psychometric properties and psychometric equivalence to the original English versions (Cronbach α=.83-.98).

#### Qualitative Data

A subsample of patients (n=77) will participate in focus groups or individual semistructured interviews. Seven focus group interviews (n=35) will be conducted among the Joy Luck Academy participants to identify common themes surrounding their Joy Luck Academy experience. Approximately 4 to 6 respondents from each Joy Luck Academy cohort will be approached by the program facilitator to participate in the focus groups [48]. The focus groups will be conducted within 1 week of completion of the intervention. The community research coordinator, who has been trained in qualitative interviewing, will conduct the focus group interviews at the HCA.

Individual interviews will be conducted after the last follow-up assessment to explore the culturally specific mechanisms that may explain the potential intervention effect. In-depth semistructured individual interviews will be conducted with 42 participants (half from the control group and half from those in the intervention group who have not participated in the focus groups). Approximately 6 women from each Joy Luck Academy cohort will participate in individual interviews to reach data saturation [49]. Each interview will last 60 to 90 minutes. These interviews were designed to explore participants’ breast cancer experience, cancer management, supporting resources, concerns, and the effect of cancer on their lives and social networks. Each topic area will be evaluated to identify additional information and specific examples. Joy Luck Academy participants will also be asked about their experiences in the Joy Luck Academy and the changes they had experienced as a result of the Joy Luck Academy. The inductively and deductively designed interview topic guide was finalized through discussion with the HCA. Permission to audio record the individual interviews and focus groups will be obtained from the participants. Participants will receive an additional US $30 compensation for the interviews.

### Data Analysis

#### Deductive Data Analysis Plan

Descriptive statistics and correlations among the major variables will be computed. The internal consistency of the questionnaires’ reliability at baseline will be assessed using Stata 16.0 software (StataCorp LLC). The construct validity of the questionnaires will be assessed using confirmatory factor analysis with Stata 16.0. Appropriate techniques (eg, exploratory factor analysis) will be used to improve reliability and validity, when necessary.

The primary analytic strategy will be multilevel analyses, also referred to as hierarchical linear modeling or linear mixed-effects modeling. Specifically, for each outcome variable, the primary independent variables of interest will include the intervention condition (experimental vs control), time (postintervention and 1- and 4-month follow-ups), and the interaction between the intervention condition and time. Random subject or Joy Luck Academy cohort effects, as applicable, will be used to model the correlations between repeated measurements within subjects and between observations from subjects within Joy Luck Academy cohorts. The selection of the random-effect covariance and serial correlation structure (eg, autoregressive correlation), as appropriate, will be based on the Bayesian information criterion. Each hierarchical linear modeling for assessing aim 1 will use quality of life as the primary outcome and other specified variables as secondary outcomes, controlling for the baseline outcome. In addition, results will be reported by controlling for important covariates used in the randomization (eg, age at baseline, cancer stage, and time since treatment) [50]. We will analyze the impact of the intervention on cortisol by testing the change in diurnal profile from pre- to postintervention using linear mixed-effects modeling. Cortisol outcomes will be indicated by raw cortisol values, initial levels, slopes, and areas under the curve, following previous methods [51-54].

Moderation effects (aim 2) will be assessed by adding potential moderators and testing their interactions with the intervention condition and time, one moderator at a time, and testing the significance of the interaction effect between the moderator and the intervention (and time, as appropriate). Mediation effects (aim 3) will be assessed by examining bootstrapped 95% CI of the indirect effect of the intervention on the primary and secondary outcomes via each hypothesized mediator [55,56]. In the presence of more than one significant single mediator, we will fit multiple mediator models to evaluate the joint mediation effects of multiple mediators [55,57]. All statistical tests will be conducted at a two-sided significance level of *P*=.05. Except for the primary analysis for the quality-of-life outcome, the findings based on other analyses will be interpreted with caution because of a lack of control over the overall type 1 error rate across multiple tests. Effect sizes with 95% CIs will also be calculated for models to help characterize the magnitude and potential replicability of the findings.

#### Missing Data

Our proposed primary analysis approaches are likelihood-based, which are valid under the missing-at-random mechanism; that is, the probability that an outcome is missing depends only on the observed variables included in the model. In such cases, no imputation of missing data will be necessary. However, we may conduct a sensitivity analysis to determine the sensitivity of our primary findings to key patterns of missing data, particularly patterns that are consistent with the missing-not-at-random mechanism, using pattern-mixture models [25]. Additional approaches such as multiple imputations will be used, as necessary. Similar results from the sensitivity analyses would strengthen our study findings, whereas different results would suggest that caution should be taken when interpreting our findings.

#### Qualitative Data Analysis

The interviews will be transcribed and analyzed in Chinese to preserve the linguistic meanings and enhance the trustworthiness of the data. In the thematic analyses, we will code responses, extract broad themes, and identify subthemes [58].

## Results

Recruitment began in February 2015, and all data collection was completed by February 2019. We expect to complete data management by August 2021 and submit the study results for publication by 2022.

## Discussion

Few evidence-based psychosocial interventions designed for diverse ethnic groups of cancer survivors are available. This study is the first RCT to test a culturally sensitive social support intervention in Asian American (specifically, Chinese immigrant) breast cancer survivors. In this trial, we will determine the health benefits of a 7-week education and peer mentorship intervention on quality of life, depressive symptoms, positive affect, and other outcomes in Chinese American breast cancer survivors immediately after the intervention and at 1- and 4-month follow-up assessments. These individuals may benefit from interventions tailored to their needs for survivorship health care information [10] and social support [7]. This program has the potential to be adapted for other Asian American immigrant breast cancer survivors and eliminate unnecessary emotional and physical health disparities in cancer care.

The design of this study has both strengths and limitations. The intervention is innovative, as it is culturally relevant to an ethnic group and uses a theoretically grounded CBPR approach. As this study targets breast cancer survivors who have completed treatment, cancer patients undergoing active treatment will not be enrolled in the study, which limits its generalizability. The Joy Luck Academy is specifically designed to meet the needs of Chinese American breast cancer survivors and, therefore, may not be directly applicable to survivors of other ethnic groups. Future studies are needed to test this type of intervention in other minority groups.

If the Joy Luck Academy is demonstrated to improve the well-being of Chinese American breast cancer survivors, it may be disseminated to this population across the country.

## Abbreviations

CBPR: community-based participatory research
CES-D: Center for Epidemiologic Studies Depression
FACT-B: Functional Assessment of Cancer Therapy-Breast
FACT-G: Functional Assessment of Cancer Therapy-General
HCA: Herald Cancer Association
RCT: randomized controlled trial

## References

[1] Gomez SL, Noone A, Lichtensztajn DY, Scoppa S, Gibson JT, Liu L, Morris C, Kwong S, Fish K, Wilkens LR, Goodman MT, Deapen D, Miller BA (2013). Cancer incidence trends among Asian American populations in the United States, 1990-2008. J Natl Cancer Inst.

[2] Thompson CA, Gomez SL, Hastings KG, Kapphahn K, Yu P, Shariff-Marco S, Bhatt AS, Wakelee HA, Patel MI, Cullen MR, Palaniappan LP (2016). The Burden of Cancer in Asian Americans: A report of national mortality trends by Asian ethnicity. Cancer Epidemiol Biomarkers Prev.

[3] Stanton AL (2006). Psychosocial concerns and interventions for cancer survivors. J Clin Oncol.

[4] Ganz PA, Coscarelli A, Fred C, Kahn B, Polinsky ML, Petersen L (1996). Breast cancer survivors: Psychosocial concerns and quality of life. Breast Cancer Res Tr.

[5] Ashing-Giwa KT (2005). The contextual model of HRQoL: a paradigm for expanding the HRQoL framework. Qual Life Res.

[6] Warmoth K, Cheung B, You J, Yeung NC, Lu Q (2017). Exploring the social needs and challenges of Chinese American immigrant breast cancer survivors: a qualitative study using an expressive writing approach. Int J Behav Med.

[7] Leng J, Lee T, Sarpel U, Lau J, Li Y, Cheng C, Chang M, Gany F (2012). Identifying the informational and psychosocial needs of Chinese immigrant cancer patients: a focus group study. Support Care Cancer.

[8] Wen K, Fang CY, Ma GX (2014). Breast cancer experience and survivorship among Asian Americans: a systematic review. J Cancer Surviv.

[9] Lee S, Chen L, Ma GX, Fang CY, Oh Y, Scully L (2013). Challenges and needs of Chinese and Korean American breast cancer survivors: in-depth interviews. N Am J Med Sci (Boston).

[10] Wen K, Hu A, Ma G, Fang C, Daly M (2014). Information and communication needs of Chinese American breast cancer patients: perspectives on survivorship care planning. J Community Support Oncol.

[11] Stanton AL, Rowland JH, Ganz PA (2015). Life after diagnosis and treatment of cancer in adulthood: contributions from psychosocial oncology research. Am Psychol.

[12] Faller H, Schuler M, Richard M, Heckl U, Weis J, Küffner R (2013). Effects of psycho-oncologic interventions on emotional distress and quality of life in adult patients with cancer: systematic review and meta-analysis. J Clin Oncol.

[13] Scheier MF, Helgeson VS, Schulz R, Colvin S, Berga S, Bridges MW, Knapp J, Gerszten K, Pappert WS (2005). Interventions to enhance physical and psychological functioning among younger women who are ending nonhormonal adjuvant treatment for early-stage breast cancer. J Clin Oncol.

[14] Tabrizi FM, Radfar M, Taei Z (2016). Effects of supportive-expressive discussion groups on loneliness, hope and quality of life in breast cancer survivors: a randomized control trial. Psychooncology.

[15] Helgeson VS, Cohen S, Schulz R, Yasko J (2000). Group support interventions for women with breast cancer: Who benefits from what?. Health Psychol.

[16] Shin H, Kominski R (2010). Language use in the United States: 2007. American Community Survey Reports (ACS-12), U.S. Census Bureau.

[17] Lu Q, You J, Man J, Loh A, Young L (2014). Evaluating a culturally tailored peer-mentoring and education pilot intervention among chinese breast cancer survivors using a mixed-methods approach. Oncol Nurs Forum.

[18] Kagawa-Singer M (2000). Improving the validity and generalizability of studies with underserved U.S. Populations expanding the research paradigm. Ann Epidemiol.

[19] Minkler M, Wallerstein N (2008). Community-Based Participatory Research for Health: From Process to Outcomes 2nd Edition.

[20] Tanjasiri SP, Kagawa-Singer M, Nguyen T, Foo MA (2002). Collaborative research as an essential component for addressing cancer disparities among Southeast Asian and Pacific Islander women. Health Promot Pract.

[21] Viswanathan M, Ammerman A, Eng E, Garlehner G, Lohr KN, Griffith D, Rhodes S, Samuel-Hodge C, Maty S, Lux L, Webb L, Sutton SF, Swinson T, Jackman A, Whitener L (2004). Community-based participatory research: assessing the evidence. Evid Rep Technol Assess (Summ).

[22] Lu Q, Yeung NC, You J, Dai J (2016). Using expressive writing to explore thoughts and beliefs about cancer and treatment among Chinese American immigrant breast cancer survivors. Psychooncology.

[23] Warmoth K, Yeung NC, Xie J, Feng H, Loh A, Young L, Lu Q (2020). Benefits of a psychosocial intervention on positive affect and posttraumatic growth for Chinese American breast cancer survivors: a pilot study. Behav Med.

[24] Zhou M (2009). Contemporary Chinese America: Immigration, Ethnicity, and Community Transformation.

[25] Little R, Rubin D (2002). Statistical Analysis With Missing Data, 2nd Edition - Wiley Series in Probability and Statistics.

[26] Pocock SJ, Simon R (1975). Sequential treatment assignment with balancing for prognostic factors in the controlled clinical trial. Biometrics.

[27] Andersen BL, Golden-Kreutz DM, Emery CF, Thiel DL (2009). Biobehavioral Intervention for Cancer Stress: Conceptualization, Components, and Intervention Strategies. Cognit Behav Pract.

[28] Cancer information in other languages. American Cancer Society.

[29] Stephenson M (2000). Development and validation of the Stephenson Multigroup Acculturation Scale (SMAS). Psychol Assess.

[30] Wan C, Zhang D, Yang Z, Tu X, Tang W, Feng C, Wang H, Tang X (2007). Validation of the simplified Chinese version of the FACT-B for measuring quality of life for patients with breast cancer. Breast Cancer Res Treat.

[31] Brady MJ, Cella DF, Mo F, Bonomi AE, Tulsky DS, Lloyd SR, Deasy S, Cobleigh M, Shiomoto G (1997). Reliability and validity of the Functional Assessment of Cancer Therapy-Breast quality-of-life instrument. J Clin Oncol.

[32] Boey KW (1999). Cross-validation of a short form of the CES-D in Chinese elderly. Int J Geriat Psychiatry.

[33] Radloff LS (1977). The CES-D Scale. Appl Psychol Meas.

[34] Wong R, Wu R, Guo C, Lam JK, Snowden LR (2012). Culturally sensitive depression assessment for Chinese American Immigrants: Development of a comprehensive measure and a screening scale using an item response approach. Asian Am J Psychol.

[35] Watson D, Clark LA, Tellegen A (1988). Development and validation of brief measures of positive and negative affect: The PANAS scales. J Personal Soc Psychol.

[36] Chandran V, Bhella S, Schentag C, Gladman DD (2007). Functional assessment of chronic illness therapy-fatigue scale is valid in patients with psoriatic arthritis. Ann Rheum Dis.

[37] Cohen S, Kamarck T, Mermelstein R (1983). A global measure of perceived stress. J Health Soc Behav.

[38] Sephton S, Sapolsky RM, Kraemer HC, Spiegel D (2000). Diurnal cortisol rhythm as a predictor of breast cancer survival. J Natl Cancer Inst.

[39] Smyth JM, Ockenfels MC, Gorin AA, Catley D, Porter LS, Kirschbaum C, Hellhammer DH, Stone AA (1997). Individual differences in the diurnal cycle of cortisol. Psychoneuroendocrinology.

[40] Kirschbaum C, Hellhammer DH (1994). Salivary cortisol in psychoneuroendocrine research: Recent developments and applications. Psychoneuroendocrinology.

[41] Gagné M (2003). The role of autonomy support and autonomy orientation in prosocial behavior engagement. Motiv Emot.

[42] Mak WW, Cheung RY (2010). Self-stigma among concealable minorities in Hong Kong: conceptualization and unified measurement. Am J Orthopsychiatry.

[43] Merluzzi TV, Nairn RC, Hegde K, Martinez Sanchez MA, Dunn L (2001). Self-efficacy for coping with cancer: Revision of the Cancer Behavior Inventory (version 2.0). Psychooncology.

[44] Sherbourne CD, Stewart AL (1991). The MOS social support survey. Soc Sci Med.

[45] Yu DS, Lee DT, Woo J (2004). Psychometric testing of the Chinese version of the medical outcomes study social support survey (MOS-SSS-C). Res Nurs Health.

[46] Lai JC, Cheung H, Lee W, Yu H (1998). The utility of the revised life orientation test to measure optimism among Hong Kong Chinese. Int J Psychol.

[47] Lu Q, Zheng D, Young L, Kagawa-Singer M, Loh A (2012). A pilot study of expressive writing intervention among Chinese-speaking breast cancer survivors. Health Psychol.

[48] Krueger RA (1994). Focus Groups: A Practical Guide for Applied Research. 2nd edition.

[49] Guest G, Bunce A, Johnson L (2006). How many interviews are enough?. Field Methods.

[50] Kahan BC, Morris TP (2012). Improper analysis of trials randomised using stratified blocks or minimisation. Stat Med.

[51] Miller R, Stalder T, Jarczok M, Almeida DM, Badrick E, Bartels M, Boomsma DI, Coe CL, Dekker MC, Donzella B, Fischer JE, Gunnar MR, Kumari M, Lederbogen F, Power C, Ryff CD, Subramanian S, Tiemeier H, Watamura SE, Kirschbaum C (2016). The CIRCORT database: Reference ranges and seasonal changes in diurnal salivary cortisol derived from a meta-dataset comprised of 15 field studies. Psychoneuroendocrinology.

[52] Pruessner JC, Kirschbaum C, Meinlschmid G, Hellhammer DH (2003). Two formulas for computation of the area under the curve represent measures of total hormone concentration versus time-dependent change. Psychoneuroendocrinology.

[53] Cash E, Sephton S, Chagpar A, Spiegel D, Rebholz W, Zimmaro L, Tillie J, Dhabhar F (2015). Circadian disruption and biomarkers of tumor progression in breast cancer patients awaiting surgery. Brain Behav Immun.

[54] Ho RT, Fong TC, Chan CK, Chan CL (2013). The associations between diurnal cortisol patterns, self-perceived social support, and sleep behavior in Chinese breast cancer patients. Psychoneuroendocrinology.

[55] MacKinnon D (2008). Introduction to Statistical Mediation Analysis.

[56] Preacher KJ, Hayes AF (2004). SPSS and SAS procedures for estimating indirect effects in simple mediation models. Behav Res Methods Instru Comput.

[57] Preacher KJ, Hayes AF (2008). Asymptotic and resampling strategies for assessing and comparing indirect effects in multiple mediator models. Behav Res Methods.

[58] Braun V, Clarke V (2006). Using thematic analysis in psychology. Qual Res Psychol.

